# Information and deliberation in the Covid-19 crisis and in the climate crisis: how expertocratic practices undermine self-government and compliance

**DOI:** 10.1057/s41269-022-00267-2

**Published:** 2022-10-21

**Authors:** Julian Frinken, Claudia Landwehr

**Affiliations:** Johannes Gutenberg-University Mainz, Mainz, Germany

**Keywords:** Covid-19 pandemic, Climate crisis, Expertocracy, Emergency powers, Deliberation

## Abstract

At the beginning of the Covid-19 pandemic, democracy’s promise to enable well-informed, responsible decisions gained almost unprecedented appeal. At this stage, many European governments mainly deferred to expert judgment. This is what some experts and activist groups occasionally call for in the case of an even more severe global crisis: the climate crisis. But where citizens are asked to more or less blindly follow the lead of expert judgments, politics takes what Lafont (Democracy without shortcuts: a participatory conception of deliberative democracy. Oxford University Press, Oxford, 10.1093/oso/9780198848189.001.0001, 2020) calls an ‘expertocratic shortcut’. In the first part of this paper, we delineate the perceptions of threat that characterize these two cases and that can lead to expertocratic temptations. We point out that shortcuts to democratic decisions not only constitute dead ends, but can also be used to reinforce existing power structures. In the second part, we show how and why such shortcuts are sociologically likely to cause alienation and reactance, as accountability is lost and the rationale for decisions cannot be retraced. We conclude that if a democratic system is to live up to its promise of rationality, legitimate expert involvement has to meet three requirements: political mandate and control, transparency of uncertainty and expert disagreement, linkage to inclusive and effective citizen deliberation.

## Introduction

At the beginning of the Covid-19 pandemic, democracy’s promise to enable well-informed, responsible decisions gained almost unprecedented appeal. In the face of shock and extreme uncertainty, the criteria for success—limiting infections and fatalities while keeping economic damages low—seemed uncontroversial, und the challenge merely one of selecting the right strategies to achieve these ends. Struggling to demonstrate liberal democracy’s superiority over authoritarian regimes like China and populist governments like Donald Trump’s in the US, many European leaders vouched to defer to expert judgment in their decision-making. For example, the Swedish government followed Anders Tegnell’s advice to pursue a strategy of herd immunity, whereas the German government in the first phase of the pandemic preferred to seek advice from virologists and epidemiologists advocating a restrictive ‘No-Covid’-strategy. However, in asking citizens to follow their lead in deferring more or less blindly to expert judgments, governments have taken what Cristina Lafont (2020) calls an ‘expertocratic shortcut’. That is, they have tried to avoid the long and arduous road of inclusive will-formation and justification by asking citizens to just accept decisions by those who supposedly know better. Expertocratic shortcuts to decision-making can be very much in demand in emergency situations. However, they are harmful to democratic systems in two main respects: First, by undermining democratic self-government, they tend to reinforce existing power relations. Second, expertocratic justifications and shortcuts to decision-making can validate reliance on individual opinion leaders and the purely instrumental use of evidence, thereby creating a resonance base for reactant, populist mobilization. The relevance of this problem becomes even clearer when we consider a second crisis that is also characterized by the perception of a major threat and the need for informed decision-making: the climate crisis. Particularly in view of the fact that our societies will have to grapple with this crisis for much longer than with the Covid-19 crisis, we should not give in to the tendency toward expertocratic governance that has become visible in the pandemic.

Our paper starts out by delineating the perceptions of threat and high uncertainty that characterize the situations that are perceived as states of emergency in the cases of both the Covid-19 and the climate crisis. It is apparent that the perception of climate change as a rather gradually unfolding process is becoming increasingly unfounded (“Covid-19 and the climate crisis: perceptions of threat” section). Consequently, the framing of a ‘climate emergency’ is gaining ground. This is reasonable, but can also form a basis for justifying expertocratic shortcuts. We go on to describe respective temptations for the two cases (“Expertocratic temptations in the Covid-19 pandemic and the climate crisis” section) and point out why shortcuts to democratic decisions ultimately constitute dead ends (“Why shortcuts to democratic decisions are dead ends” section). Drawing primarily on the German experience, we illustrate the societal consequences of expertocratic government and show how expertocratic justifications and shortcuts to decision-making can validate reliance on individual opinion leaders and the purely instrumental use of evidence, thereby creating a resonance base for reactant, populist mobilization (“Societal consequences of expertocratic government” section). We thus conclude that eventually, expertocratic practices not only undermine democratic self-government, but also risk compliance with laws and regulations. In “Three requirements for a democratic use of expertise” section, we thus finally identify three requirements for legitimate expert involvement in political decision-making, arguing that while there is room and need for experts and expertise in a deliberative system, their authority and mandate must result from inclusive meta-deliberative processes and must be contestable and transparent.

## Covid-19 and the climate crisis: perceptions of threat

Expertocratic emergency powers can be justified with reference to a perceived threat, an emergency situation requiring rapid action and leaving no time for lengthy democratic procedures and discourses. Both the Covid-19 pandemic and the climate crisis can be understood as emergencies of this kind, taking place on a global scale. They appear as dangerous natural disasters that require rapid and informed policy responses to prevent even more severe impacts.^1^ At first glance, however, there also appear to be major differences between the perceptions of these two crises and the policy responses they provoke. In Germany and several other European countries, the pandemic was quickly perceived as an emergency and responded to in a technocratic-rational manner (Landwehr and Schäfer 2021).

Climate change, in turn, has been on the agenda for decades. Its potential danger was presented to the global public as a scientific consensus as early as 1979 at the first World Conference on Climate Change (Gupta 2014, p. 41). Yet, it seems that it has so far been perceived as less pressing by large parts of the public and by political decision-makers.

As we show in this section, some key factors that explain the different threat perceptions and responses are becoming increasingly irrelevant. The climate crisis is more and more taking on the character of an emergency situation and is more often being communicated as such. As a result, calls for expertocratic governance become louder. Our argument boils down to the point that we must not give in to these: not only do they turn out to be problematic shortcuts to democratic decision-making, but the long-term nature of the climate crisis makes their negative consequences for democracy even more far-reaching.

In three key respects, the Covid-19 pandemic and the climate crisis seemed to unfold in different ways, which, at first sight, may help to explain divergent threat perceptions and responses. First, with respect to climate change, it is often argued that the effects of the ongoing temperature rise unfold gradually over a longer period of time due to the inertia of the climate system (Victor 2011, p. 40). Future generations will thus be much more affected by current political decisions than people living today. This may be especially true for inhabitants of the northern hemisphere, who have been much less severely impacted so far. In contrast, the emergence of SARS-CoV-2 type occurred in the form of a sudden rupture, following its rapid spread across a globalized planet. The dangers quickly became omnipresent in the media through images of ventilated patients in overcrowded hospitals, and were later felt by many in their own lives as well. Second, and relatedly, the effects are distributed unevenly in geographical terms. While societies in the global North are less affected by the consequences of the global rise in temperature and are potentially better able to adapt to it because of greater material resources, global warming affects societies in the global South much more severely. Some Pacific Island states are even threatened in their very existence (Fletcher 2009). In the case of the Covid-19 pandemic, the situation was just the opposite: After the virus spread in China and Iran, Europe, at a very early stage, became the epicenter of the pandemic. Malm (2020, pp. 20–23) regards this as a reason for the decisive action taken by Western governments, which they are lacking in the case of the climate crisis. Third, the effects of the climate crisis take very different forms, such as extreme weather, the decline of habitable conditions and fertile soils, and the destruction of entire ecosystems on land and in the sea. It is impossible to point a finger at the multifaceted phenomenon of the climate crisis. Literary scholar and publicist Timothy Morton coined the term ‘hyperobjects’ (2013) for phenomena that span time and space to such an extent that they are difficult for humans to fully understand or experience. A new virus causing a particular pandemically spreading disease, by contrast, is much more tangible.

While these differences may go some way in explaining why the threat of the climate crisis is seen by parts of the public and among policy makers as less immediate than the threat posed by SARS-CoV-2, these differences are becoming more and more insignificant as the climate crisis progresses: The purported gradualness of climate change is increasingly being challenged by research indicating that it appears to be occurring more rapidly than previously thought (Ripple et al. 2020, p. 9) and that certain tipping points could already be causing uncontrollable disruptions to the climate system (Lenton et al. 2019). Ever more frequent extreme weather events, such as forest fires, droughts, and heavy rains cause abrupt disruption even in the global North, increasingly shattering what still appears to be a widespread perception of slowly advancing climate crisis (see Malm 2020, p. 18). The geographic imbalance of affectedness is mitigated by increasingly tangible climate change impacts in countries of the global North. The fact that these take place as concrete events, such as heat waves, crop failures, and floods, makes the ‘hyperobject’ of climate change take on concrete, albeit multiple, forms, thus generating political pressure for action with each successive incident.

Still, there is one central difference between the two crisis that also influences threat perceptions: While no one benefits from the spread of SARS-CoV-2, this is not the case for the externalization of costs to future generations with greenhouse gas emissions (Malm 2020, p. 29). Thus, another key factor of political inaction regarding the climate crisis is tactics of delay by powerful lobby groups, especially from the fossil fuel industry. For decades, lobbyists have been working against scientifically well-established findings, repeatedly trying to cast doubt on the scientific consensus of anthropogenic climate change through targeted campaigns of an ‘organized denial’ (Jamieson 2017, p. 81; cf. Oreskes and Conway 2010). These tactics of delay have successfully helped to reduce the threat perception in large parts of the public.

Against such false appeasements and in light of the actually increasing threat, social movements in particular have succeeded in recent years in raising public awareness of the climate crisis, deliberately using emergency rhetoric. For example, the Fridays for Future movement uses the term ‘climate emergency’ in its public communication (Fridays for Future n.d.). The same applies to the global climate justice movement Extinction Rebellion, whose logo, a pictogram of an hourglass, emphasizes the aspect of lack of time. Increasingly, this rhetoric is also used with appellative intent in the scientific community (Lenton et al. 2019; Ripple et al. 2020). The goal of such framing is to get governments and the public to view the climate crisis as an emergency and to ‘[u]nite behind the science’ (Fridays for Future 2019) in order to take quick political action based on scientific evidence.

Now that the Covid-19 pandemic seems largely contained in large parts of the world and the climate crisis is coming more to the fore, many activists and scientists are calling for lessons to be learned from the pandemic for the fight against the climate crisis (see e.g., Malm 2020). We argue that one core lesson consists in not falling for expertocratic temptations, although and precisely because they become more relevant with an ever-stronger emergency framing of the climate crisis. The following section goes on to illustrate these tendencies for both cases.

## Expertocratic temptations in the Covid-19 pandemic and the climate crisis

As the novel and potentially deadly SARS-CoV-2 virus spread across the globe at a rapid pace in the early 2020s, the world's governments had to act quickly. Many European governments therefore demonstratively relied on a policy guided by expert judgments. While the Swedish government followed Anders Tegnell's advice and initially indicated a strategy of herd immunity, German Chancellor Angela Merkel stated that decisions on containment strategies would follow the recommendations of leading virologists, such as Christian Drosten of the Berlin Charité, and the Robert Koch Institute, a government agency for disease control. The German Federal Ministry of Finance also acted in particularly close consultation with economists when it came to putting together economic stimulus packages. At the same time, legislative procedures were weakened by adjustments to a new Infection Protection Act (*Infektionsschutzgesetz*) in favor of administrative order rights (Schwanholz 2021, p. 67), and Chancellor Merkel spoke out against ‘discussion orgies’ among the state governments in an internal conversation (Fiedler and Starzmann 2020). Thus, while democratic procedures and discourses were marginalized on the part of the government, experts in the meantime appeared to determine the government’s course to a substantive degree.

Simultaneously, virologists in particular found themselves in the focus of public attention in this early phase of the pandemic. They dominated talk shows, provided information and explanations in podcasts and at press conferences. The increasing overlap of the spheres of politics and science was commented on in two of the most important German newspapers, *Die Zeit* and *Der Spiegel*, by envisioning Christian Drosten as German Chancellor (Hornig et al. 2020; Lau 2020). In the U.S., the fantasy of replacing the president with his disliked adviser Anthony Fauci lent itself to expression on ‘Fauci for President’ T-shirts. The longing for expertocratic governance is in part understandable against the backdrop of the Trump administration’s erratic and irrational Corona policies. In Germany, too, a majority of the population supported the strict measures in the beginning of the pandemic and support for the CDU-led government that adopted expertocratic strategies soared (Schraff 2020).

In the case of the Covid-19 pandemic, governments themselves, with support from large parts of the public, thus gave in to the expertocratic temptation. In the climate policy discourse, by contrast, it is individual voices from science and in parts the public communication of social movements that reveal such a temptation. In general, expertocratic demands have a certain tradition in environmental policy thinking. Linked to the diagnosis that democratic systems are not capable of dealing with serious environmental problems, such demands appeared early on, for example in Heilbroner (1974) and Ophuls (1977). Most notably, natural scientist James Lovelock questioned the performance of democratic systems by drawing a war analogy: ‘climate change may be an issue as severe as war. It may be necessary to put democracy on hold for a while’ (Lovelock quoted in Hickman 2010).^2^ For Shearman and Smith (2007, p. 85), it is above all the short-term thinking and the self-interest of representatives, who almost exclusively have their re-election in mind, that disqualifies liberal democracy as an effective system for halting climate change. In the current debate, the Fridays for Future slogan ‘follow the science’ expresses understandable frustration with many years of political denial of scientific findings and the above-mentioned delaying tactics of powerful lobbies. It rightly urges a policy based on scientific facts. But in its literal reading, it forgets that science cannot lead politically or even exercise legitimate political power.

Besides the time constraints associated with the emergency situation, the central argument that justifies the transfer of political authority to experts in at least a circumscribed realm is that the criteria for ‘correct’ political decisions are clear anyway and that it is now simply a matter of implementing them on the basis of adequate information. At the beginning of the Covid-19 pandemic, the primary concern had to be limiting infections and deaths while at the same time containing the economic damage. In the case of the climate crisis, the top priority is to reduce emissions of greenhouse gases as quickly as possible and to ultimately bring them to a halt.

For a short and limited time, expertocratic emergency powers may certainly be justified in order to avert greater damage, especially to the foundations and stability of the democratic constitution itself. However, the risks associated with demanding and implementing such emergency powers become apparent when considering that the climate crisis is a permanent crisis that cannot simply be stopped or reversed by adopting a specific set of ‘right measures’. Instead, it is much more a matter of preventing the worst catastrophe and, beyond that, dealing with the new conditions—an adaptation that can hardly be achieved without drawing on the evidence and support base of the democratic demos itself. More importantly, from the perspective of a normative democratic theory that understands democratic governance as self-government, reducing political decision-making in the name of ‘truth-tracking’ in order to achieve solutions that are rational in a scientific sense constitutes an illegitimate ‘expertocratic shortcut’ (Lafont 2020, p. 78).

## Why shortcuts to democratic decisions are dead ends

According to Bogner (2021), the expertocratic temptations described above are only symptoms of a more general trend towards an *epistemicization of the political* (Bogner 2021), which is reflected in everyday politics by an increasingly comprehensive delegation of political decisions to non-majoritarian expert bodies. He analyzes and describes the tendency of an *epistemicization of the political* as the idea that reasonable and responsible politics is possible solely on the basis of superior scientific knowledge (Bogner 2021, p. 114). In practice, this idea is reflected in an expertocratization of politics that bypasses broad and democratic procedures of will-formation by transferring decisions to specialized bodies. From this perspective, crises such as the Covid-19 pandemic or climate change are understood primarily as epistemic problems. It is undeniable that dealing with such crises requires a particular level of specialized knowledge and thus comprehensive scientific policy advice. However, the assumption that experts can therefore also make better political decisions is misguided in several ways.^3^

First, the demand for a transfer of decision-making power to scientific authorities is based on a simplistic and ultimately false assumption of objectivity that disregards the factor of scientific uncertainty (Saretzki 2011, p. 61). The expertocratic modeling of the interface between science and politics can be described as *linear*: According to this model, scientific findings determine the right political measures, which are legitimized precisely by the fact that they are supposedly objectively right (van der Sluijs et al. 2010, p. 410). But this postulation of objectivity underestimates the factor of uncertainty in science: Even if the fact of anthropogenic climate change can be described as a scientific consensus, there is no unanimity within the research community either about the concrete extent of the expected consequences or about the measures to be taken, i.e., the action that follows from the knowledge. Consequently, there is no objective best practice that some council of scientists could decide on with unanimity beyond all doubt (cf. Saretzki 2011, p. 61). At the beginning of the Covid-19 pandemic, there was also a high degree of uncertainty. While the learning curve has been exceptionally steep, even enabling the development and approval of a vaccine within less than a year, considerable and reasonable disagreement remains within the scientific community. Disagreements concern, for example, the long-term health effects of infections in different age groups and the benefits of vaccinating younger children. Moreover, social and economic consequences of containment measures remain to be explored.

Second, the assumption that scientific methods can provide answers to political questions is mistaken. When it comes to weighing benefits of disease control against social and economic damages, it is clear that no ‘scientific’ calculus could do the job: political decisions require societal and democratic value judgments to trade off conflicting interests and concerns. They regularly produce winners and losers since they deal with the fair distribution of specific goods and burdens. Scientific claims cannot constitute answers to the normative questions political decisions have to address. Moreover, an authority of experts in the decision-making process on such normative questions would undermine the democratic legitimacy of the respective decision. This applies not only to the formulation of societal aims, but also to the choice of instruments with which these aims are to be achieved. Even assuming that there was a societal consensus on the desired aims and that experts were only entrusted with the task of determining the right means to achieve them, there are often functionally equivalent ways with diverging practical implications to achieve the same goal (Saretzki 2011, p. 61). One might ask, for example, what proportions of the Paris Agreement’s 1.5–2 °C target should be achieved by consistently shutting down coal-fired power plants, banning individual passenger transport, eliminating factory farming, or applying geoengineering technologies. While a consideration of such options must in principle be informed by scientific evidence about their effects, it involves questions that are, in Jürgen Habermas’ typology, in their essence ethical–political ones (Habermas 1992, p. 198). Whether one prefers, for example, to leave natural resources untouched or to manipulate the climate system by technical means in order to achieve the same quantitative goal of a limited temperature increase is also an expression of one's own moral orientation toward the world. Scientific expertise alone simply cannot provide definitive answers to questions that concern society as a whole and ultimately aim at moral issues and ethical–political questions of a shared good life (Fischer 2017, p. 272). Answering these questions cannot be done from an objective point of view, but requires a democratic discourse in which, on the one hand, there is no normative justification for a special position of experts (ibid.), and on the other hand, no better answers can be expected epistemically unless all relevant perspectives are taken into account.

The various strategies for tackling climate crisis not only involve moral issues of the kind mentioned above, but they also reflect manifest conflicts of distribution. The alternative paths that may be taken all involve costs and benefits for different actors. The question of how these are to be allocated must be addressed through democratic and inclusive processes that weigh different considerations of fair distribution. The expertocratic idea of welfare-maximizing political decisions is blind to the very circumstances of politics, which consist in the fact that ‘we disagree both about the right and the good, yet nonetheless require a collective decision on these matters’ (Weale 2007, p. 5).

An epistemic conception of democracy and expertocratic government strategies therefore offer no answers to political distribution conflicts between different actors, which are often at the heart of the problem. This omission of distributional issues, however, does not make conflicts disappear, but only obscures them, thus reinforcing existing power structures in the most problematic way. This becomes clear when state actors themselves blur the boundaries between science and politics in their global climate policy efforts: In 2009, the Copenhagen Accord set the goal of limiting the global temperature increase to 2 °C. According to the Accord, it is ‘the scientific view that the increase in global temperature should be below 2 °C’ (UNFCCC 2010, p. 5). However, this statement is problematic in two respects: First, there was and is no scientific consensus on a very concrete number (Blue 2015, p. 158), and second, every number in this context represents a normative target, which in its consequences produces winners and losers. The mode for formulating such a common goal can therefore not be a scientific one at all, but is always a political one that faces competing interests and values. Conflicting interests were only disguised in the case of the 2 °C target, hiding an imbalance of power in the decision-making process: the target in fact expressed the interest of the richer countries in the more northern latitudes, which are less likely to experience the immediate effects of climate change, while many Pacific Island Countries see their literal demise cemented in a 2 °C rise in global temperatures (Fletcher 2009). By politicizing the target, the latter eventually managed to get the 1.5 °C target included as a more ambitious aspiration in the 2015 Paris Agreement (Falkner 2016, p. 1114).

How contingent political choices are frequently being defended as beyond contestation under the guise of scientific objectivity, with a rhetoric of no alternatives strengthening existing power relations instead of putting them up for disposition also became apparent in the Covid-19 crisis. Given the disproportionately greater negative impact of the pandemic and its containment measures on already disadvantaged groups, a democratic discourse on its distributional effects remains an urgent desideratum. As in the case of the climate crisis, many governments selected measures that caused a particular strain on younger generations, those in precarious living and working conditions and on women in care relationships—a result that could have been predicted from the underrepresentation of these groups in parties and parliaments. In Germany, the observation that car dealers were allowed to open before schools caused some outcry and derision in the first wave of the pandemic, but did not prevent governments from closing schools again for more than six months throughout the second and third wave. As examples like neighbouring France show, the same degree of containment could well have been achieved by different combinations of measures, such as stricter curfews or obligatory requirements to work from home—measures that would of course have taken a higher toll on different groups and interests.

In sum, the epistemicization of the political thus obscures two aspects of political reality: First, that there is uncertainty and legitimate disagreement in science and secondly, that all political decisions entail value and distributional conflicts that must be negotiated democratically. In the next section, we argue that concealing these aspects of political decision-making behind expertocratic rule is not only normatively problematic, but also practically unsustainable, as it can cause harmful reactance to political measures, undermine trust in science and promote populist mobilization.

## Societal consequences of expertocratic government

In 2020, Germany seemed exceptionally successful in containing the Covid-19 pandemic. In fact, German success in the first wave was probably facilitated by the timely and determined implementation of lockdown measures and by clear and unambiguous political communication that highlighted the infectiousness of the virus and the severity of symptoms especially among the elderly population. In the summer of 2020, daily infection numbers were down and a return to an almost normal social life was possible for a majority of people. At the same time, success in containing the pandemic lead to seemingly paradox reactions within political elites and the public sphere. Within elites, complacency with regard to the capacity of the political system and administrative structures to deal with major challenges seemed to take hold. In the public sphere, one of the most visible and potent protest movements against Covid-containment measures emerged—despite the fact that Germany had managed to contain the disease with comparatively moderate measures and the impression that the pandemic was more or less over.

The German ‘Querdenker’-movement draws support from and can mobilize protesters from a rather diverse spectrum of social groups and backgrounds: rightwing extremists and Q-Anon supporters, anti-vaxxers and conspiracy theorists, but also the anthroposophy scene and non-medical healers (Koos and Binder 2021). However, demonstrations were also attended by small shop-owners, innkeepers or showmen fearing for their businesses. The fact that the German vaccination campaign has been stalling at around 75% is partly due to the influence of this movement, which has taken to initiating demonstrations across the world (Knaus and McGowan 2021). The alliances behind 2021 protests by truck drivers in Canada and Australia were similarly diverse as the Querdenker-movement and seemed to be partly driven by the same communication channels. But what are the psychological dynamics behind the success of these movements?

It seems that after the success of containing the first Covid wave, political communication highlighting the risks of infection and numbers of potential fatalities could no longer induce the same level of fear as in the early days of the pandemic. Messages that promoted compliance in the first wave now sparked reactance, especially among groups that perceived politics as neglecting their interests and concerns—but doing so in the name of science and morals that were presented as uncontestable. In consequence, not so much the selection of containment measures, but the very diagnosis of an emergency justifying these was contested. The resulting politicization of expertise and scientific findings meant that scientific judgments used by the government to justify their policies were confronted with counter-judgments. Counter-judgments came partly from renowned experts arriving at different risk assessments or advocating different measures as effective and partly from outright science deniers and conspiracy theorists. As Alexander Bogner has pointed out, the science denial movement should not be dismissed as one of lunatics, but tells us something about the dark sides of the knowledge society, as it gains salience where an epistemicization of the political denies the existence of alternatives (Bogner 2021, pp. 97–98). Where expertise and science become politicized, the differences between facts and opinions and between dissenting experts and conspiracy theorists are blurred on both sides of the struggle. Whereas science deniers shared ‘alternative facts’, those advocating a ‘zero-Covid’ strategy in the name of science did no longer recognize the difference between reasonably disagreeing voices and those of outright Covid and science deniers. Disregarding scientific uncertainty and legitimate disagreement can thus give way to both, expertocratic governance and science denial (Moore and MacKenzie 2020).

In essence, the politicization of expertise must be seen as the result of a political strategy that has been increasingly applied over the last decades, but gains particular traction in situations of crisis and emergency (Schäfer and Zürn 2021): executives and non-majoritarian expert bodies take decisions behind closed doors and then present them to the public as a reasonable consensus without alternatives, bypassing parties and parliaments. As a result, democratic discourses are stifled and dissenting voices dismissed as irrational, thereby fertilizing the resonance base for populist mobilization. As Schäfer and Zürn conclude, authoritarian populists, who, where in power, failed spectacularly at managing the pandemic, might ultimately benefit from the perception of irresponsive government by elites that has increased especially among marginalized groups (ibid. 164).

## Three requirements for a democratic use of expertise

As experiences in the Covid crisis have shown, decisions taken by way of expertocratic shortcuts will ultimately lack compliance because the reasons behind them are not transparent to and shared by those subjected to them: democratic citizens. If reasons are not publicly justified and critically assessed, they cannot be translated into motives to accept and comply with decisions. As numerous authors have convincingly argued, the *epistemicization* of democratic politics through expertocratic shortcuts fertilizes a resonance base for authoritarian populists, who draw on perceptions of irresponsiveness, especially among disadvantaged groups (Schäfer and Zürn 2021; Lafont 2020; Bogner 2021). Does this mean that expertise and experts are a problem for contemporary democracies and that their influence on policy-making should be limited? On the contrary: the demand for information increases in an ever more complex world and citizens rightly expect representatives to take decisions on the basis of a thorough assessment of the best available evidence. More importantly, the ideal of democracy not only entails a promise of egalitarian self-determination but also one of rationality. Citizens do expect legislatures and governments to solve societal problems and do, on the whole, expect democracies to promote liberty, welfare and justice to a higher degree than autocracies can. The central question is thus: *How can democracy live up its promise of rationality without betraying its promises of self-determination and equality?* In light of the challenges ahead, we see three necessary, but not sufficient requirements democratic government has to meet to base decisions on necessary expertise without sacrificing legitimacy and sustainability:

### Democratically mandating and controlling political expert bodies

Charged by governments or legislatures, non-majoritarian expert bodies can enable a deliberative assessment of information and arguments relevant to public policy-making. Moreover, they tend to enjoy considerable support in public opinion (Bertsou and Caramani 2022), which is most likely due to the fact that experts are expected to be ‘disinterested’, i.e., to have no own stakes in the decision at hand and seek to maximise a common good. To give these bodies a legitimate role in decision-making, however, institutional design needs to be democratized. In other words: the set-up, mandating and design of an expert body must itself be the result of an inclusive and democratic decision-making process. Such democratic institutional design processes should be meta-deliberative: they should be based on deliberation about who should deliberate and decide how and when.^4^

To meet criteria of democratic institutional design, the delegation of parts of the democratic decision-making process to experts must be based on a clear majoritarian mandate from elected representatives. At the same time, the design of non-majoritarian expert bodies (as much as that of any given decision-making procedure or institution), in particular by way of the selection of members from different disciplinary backgrounds or ‘schools of thought’, always constitutes an institutionalization of specific norms that promote some interests more than others. Against this background, it seems a dubious move to simply charge a standing expert committee with a new task, as was done in Germany when the government charged the Leopoldina, a somewhat obscure body of eminent academics, with the development of a strategy for re-opening schools and businesses. Instead, the selection of experts for a committee charged with far-reaching recommendations should be publicly justified.

Assuming that the norms inscribed into non-majoritarian bodies must be coherent with societal values for their decisions to be accepted (Landwehr and Klinnert 2015), different countries may well select different sets of experts and implement different decision-rules. In any case, however, institutional design needs to ensure ‘throughput legitimacy’ (Schmidt 2013) by ensuring transparency and accountability. Moreover, both the act of delegation and the design of expert bodies charged with recommendations must publicly be regarded as contestable and reversible. Non-majoritarian expert bodes can only have a legitimate role in democratic decision-making processes where they are subject to scrutiny from a critical public sphere.

Expert bodies in the Covid crisis, as argued above, often lacked a clear majoritarian mandate (which should have been provided by legislatures rather than executives) and their role in decision-making was insufficiently transparent and accountable. Expert bodies to play a role in addressing climate change and in developing strategies to combat it will require a stronger democratic mandate, will have to ensure throughput legitimacy and to invite discussion and contestation in order to garner support for their recommendations.

### Making uncertainty and disagreement among experts transparent

In the previous section, we argued that both the expertocratic shortcut and outright science denial are based on the same misunderstanding: the disregard of scientific uncertainty and legitimate disagreement (Moore and MacKenzie 2020). If the instrumental use of scientific knowledge as well as science denial are to be prevented, scientific expertise must not only be communicated in a comprehensible way, but potential uncertainties and disagreements among experts have to be made transparent. Moore and MacKenzie (2020) identify the publication of minority reports in expert committees as one institutional mechanism through which this could be accomplished. Along the same lines, Pamuk (2021, p. 570) suggests that the U.S. Supreme Court’s practice of the writing of dissenting opinions should also be adopted for expert bodies. This practice also serves another purpose: As Pamuk (2021) shows, the two expectations of neutrality and usefulness placed on expert commissions stand in an inherent tension with each other: To be useful, scientific advice must, to some extent, include value judgments, which tends to violate the requirement of neutrality and can thus diminish the authority and legitimacy of expertise. This dilemma can only be mitigated if expertise is exposed to greater public scrutiny, for which the publication of dissenting opinions is a prerequisite (ibid.).

Disclosure of disagreement and of value judgments makes it difficult for politicians to misrepresent selected scientific findings as implying the justification and inevitability of specific policies. It prevents a false belief in the prescriptiveness of science and promotes public discourse about what societal goals and values are worth pursuing. In addition, this openness makes it easier for decision-makers to change or correct policy measures without appearing to be inconsistent (Moore and MacKenzie 2020). Good reasons for such corrections can emerge in light of new evidence. This can be understood by the public if uncertainties have been made transparent from the beginning. The case of masking in the pandemic is a good example in this case: when, at the beginning of the pandemic, renowned experts such as Anthony Fauci saw no benefit of masks to contain the virus, they swept uncertainty and disagreement in the scientific community under the rug, thus undermining their own later recommendation to mask up (Pamuk 2021, p. 571).

### Promoting and institutionalizing inclusive and effective citizen deliberation

The kind of democratic institutional design advocated above is only possible where a vivid public sphere exists and where citizens can contest and ultimately drive public policy-making in general. As it stands, there is evidence from even the most advanced democracies that decision-making is all too often exclusive and only selectively responsive to citizens’ concerns (Schäfer and Zürn 2021). One prominent suggestion for institutional innovation has thus been the organization of democratic mini-publics—lot-based citizen forums that discuss salient topics and develop recommendations for policy-making (see Dryzek et al. 2019).

Especially where epistemic and participatory demands of democratic systems conflict, lot-based mini-publics already play an intermediary role in democratic systems. One recent example of this is the proliferation of ‘Climate Assemblies’ in many countries across Europe. They can help to ensure the representation of diverse social perspectives when it comes to weighing the political implications of complex issues and to reflecting on the aforementioned value judgments experts oftentimes have to make in crafting useful advice. This is especially important since the composition of expert bodies is usually not particularly diverse in socioeconomic terms, which can lead to blind spots regarding the real-life consequences of recommendations for certain social groups (Moore and MacKenzie 2020).

The examples of Climate Assemblies also highlight some major challenges when it comes to reconciling epistemic and participatory demands in mini-publics: In the case of the ‘Climate Assembly UK’, both participants and expert witnesses perceived the process as too short to learn or to convey all the relevant information (Elstub et al. 2021, pp. 9–10). As a result, the problematic influence of experts on participants, which arises from the epistemic imbalance between those groups, might not be sufficiently contained. In the ‘French Convention on Climate Change’, this influence was even actively promoted by some of the experts themselves in that they ‘went beyond their role, either unduly pushing for certain measures or discarding others’ (Giraudet et al. 2022, p. 10). One way to combine participatory and epistemic demands is to allow participants to appoint experts themselves, as was to some extent done in the case of the French assembly (Mellier-Wilson 2020). Another option, namely extending the whole process of a mini-public, again illustrates the tension between those two demands, as this would reduce the level of inclusion: the longer the process, the more likely it is to exclude groups that simply cannot commit to such an obligation due to their particular life-circumstances. One viable approach to these problems could be to limit the thematic scope of a single mini-public and rather hold several of these processes on more specific issues.

In general, problems also arise where the sphere of competence of a mini-public is not clearly defined and, as a result, conflicts of legitimacy arise as to which body may ultimately make binding decisions. This is what happened in the case of the French convention (Landemore 2020, pp. 118–120). As Lafont has convincingly argued, the ‘lottocratic’ idea of empowering mini-publics to take decisions constitutes just another variant of the expertocratic shortcut (Lafont 2020). If we assume that it is possible for a descriptively representative sample of the population to be comprehensively informed, to deliberate and decide on a matter, the mini-publics’ participants will no longer be representative after deliberation. Instead, they will have become experts to whose decisions the non-participating majority is asked to blindly defer to, thus violating conditions for democratic self-government. Lafont argues that such an approach inadmissibly reduces the epistemic function of deliberation as a mode of political communication to the tracking of truth. This is highly problematic because ‘another epistemic function of deliberation is disregarded, namely tracking the justifiability of the policies in question to those who must comply with them’ (ibid.: 98). If democracy is to be understood as self-government of the citizens, this justification must take place vis-à-vis all those who are subject to decisions. Otherwise, not only democratic legitimacy, but also compliance will be undermined (ibid.).

What matters to achieve both legitimacy and compliance is that citizen deliberation is horizontally encompassing and inclusive beyond the boundaries of a small mini-public and that, in a vertical direction, citizen deliberation effectively informs political decisions taken by legislatures and governments. While mini-publics offer no substitute to these representative institutions and should not be empowered to take decisions, they can still fulfill important functions for public discourses in a deliberative system. Lafont distinguishes between contestatory, vigilant and anticipatory functions (Lafont 2020, ch. 5.2). Anticipatory uses are important where the public has not yet formed opinions on important matters and depends on a variety of sources of arguments and perspectives to develop such. Contestatory uses apply where arguments of democratic minorities are inadequately heard and considered in public opinion and policy-making. Vigilant uses, finally, are important where public opinion and policy-making are in discord.

Reconsidering the Covid-19 crisis, mini-publics could have had important contestatory uses, given that the interests of parents and school children were in many countries insufficiently considered and that societal groups like shop- and restaurant-owners or showmen regarded politics as not responsive to their grievances. In the climate crisis, mini-publics could still have anticipatory functions. Although a majority of the population regards climate change as a pressing problem, opinions on strategies to combat it so far seem insufficiently formed. Given that the group of those willing to take the necessary radical steps to limit emissions still constitutes a minority position, contestatory uses of mini-publics in public discourses will also be important. If growing support for radical measures is not met with respective policies and congruence between public opinion and climate policy-making is lost, mini-publics may also become vigilant. If and when mini-publics become vigilant and receive resonance from a broader public sphere, they also highlight biases and shortcomings of the political system as such and indicate where decision-making is insufficiently responsive to at least some relevant concerns—such as climate change. From here, they can also spark larger-scale meta-deliberative discourses on institutional reforms that could improve democracies’ capacity to address pressing problems without neglecting its promise of egalitarian self-government.

Without a doubt, and as our cases illustrate, there is a great need for expertise in democratic decision-making. We have argued, however, that its use must not come with an expectation of blind deference—to use Lafont’s (2020) terminology. However, it seems unrealistic to expect that at any time all citizens agree on decisions in full awareness of all relevant evidence and arguments. There is thus certainly some need for deference in modern mass societies. But in order to *not blindly defer*, citizens must have both the capability to exercise some kind of control over their rulers and the ability to decide ‘when and how far’ they want to defer (Goodin 2020, p. 25). We consider the three requirements we have suggested here as necessary to enable citizens to exercise these capabilities.

Emergency situations, of course, strongly impede the realization of these requirements. This is particularly clear with regard to the last point, as mini-publics are very elaborate and time-consuming participation processes.^5^ There are certainly limiting cases in which a major threat puts not only a large number of lives, but also the stability and survival of a democratic system of rule itself at risk. In these cases, the temporary use of emergency powers, including the suspension of democratic procedures in favor of hierarchical and expertocratic forms of decision-making can be justified. As we have argued, however, the climate crisis, as the presently most alarming threat, is not simply an emergency situation, but must be understood as a long-term problem. Given the long time horizon for combatting the crisis and the severe conflicts that will arise over the distributions of costs for necessary adjustments and restrictions, democratic standards cannot be abandoned in climate decision-making without risking legitimacy and compliance.

## Conclusion

What can we learn from the management of the Covid-19 pandemic for the management of the climate crisis, which will be the most important task ahead of us for the rest of the century? As we have argued in this paper, the Covid crisis exemplifies how the demand not only for political, but also for epistemic authority soars in face of a major threat, and how the threat comes to be seen as at least temporarily legitimizing the use of emergency powers. However, using emergency powers—that is, suspending individual liberties and, more importantly, bypassing processes of democratic will-formation—comes at a high price to democracy. This is because the very idea that the point of democracy lies in the quest for ‘correct’, welfare-maximising solutions is a misunderstanding of the very conditions of politics: in our societies, social groups compete for resources and conflicting understandings of the good and the right that are never entirely reducible. Under these conditions, *politics is not about finding, but about constructing solutions to societal problems and challenges*. We have argued that denying the contingency and political quality that collectively binding decisions retain even in situations of emergency leads to serious legitimacy problems. Moreover, it will not be sustainable in the long run, as it is likely to lead to reactance and non-compliance among citizens. However, scientific expertise is indispensable if democracy is to deal with major challenges like climate change, and citizens expect decisions to be based on evidence and good reasons. Against this backdrop, the last section of our paper has pointed out three necessary, but not sufficient requirements for legitimate expert involvement in political decision-making: politically mandating and controlling expert bodies, making uncertainty and expert disagreement transparent and enhancing inclusive and effective citizen deliberation.

## Footnotes

1 The notion of a natural disaster however, should not obscure the fact that the causes of both crises can be traced back to human activities: In the case of the climate crisis, this is an industrialized economy based on fossil fuels. In the case of the Covid-19 pandemic, which according to current evidence was caused by transmission of the virus from an animal to humans, it is expanding urbanization and the destruction of natural habitats that increase the risk of such zoonotic disease (WHO 2020).

2 In the early stages of the Covid-19 pandemic, the war analogy was drawn again, for example, by Emmanuel Macron and also Donald Trump (Macron cited in Rose/Lough 2020; Trump cited in Cathey 2020).

3 The argument regarding climate change in this section has in a similar form been made before in Frinken (2021).

4 Parts of the argument presented in this section have been made more extensively before in Landwehr (2015).

5 However, one fruitful option could be the creation of permanent mini-public structures that can be quickly activated at any time to make use of the insights of small group citizen deliberation, even in times of acute crisis. Such models of standing and rotating mini-publics already exist in the Belgian region of Ostbelgien and the city of Paris (Chwalisz 2022; Niessen and Reuchamps 2019).
